# 3D-Printed Nanocomposite Denture-Base Resins: Effect of ZrO_2_ Nanoparticles on the Mechanical and Surface Properties In Vitro

**DOI:** 10.3390/nano12142451

**Published:** 2022-07-18

**Authors:** Ali A. Alshaikh, Abdulrahman Khattar, Ibrahim A. Almindil, Majed H. Alsaif, Sultan Akhtar, Soban Q. Khan, Mohammed M. Gad

**Affiliations:** 1 College of Dentistry, Imam Abdulrahman Bin Faisal University, P.O. Box 1982, Dammam 31441, Saudi Arabia; ali.alshaikh1997@gmail.com (A.A.A.); Abdulrahman.khattar@gmail.com (A.K.); almandil252@gmail.com (I.A.A.); majedalsaifdent@gmail.com (M.H.A.); 2 Department of Biophysics, Institute for Research and Medical Consultations (IRMC), Imam Abdulrahman Bin Faisal University, P.O. Box 1982, Dammam 31441, Saudi Arabia; 3 Department of Dental Education, College of Dentistry, Imam Abdulrahman Bin Faisal University, P.O. Box 1982, Dammam 31411, Saudi Arabia; sqkhan@iau.edu.sa; 4 Department of Substitutive Dental Sciences, College of Dentistry, Imam Abdulrahman Bin Faisal University, P.O. Box 1982, Dammam 31441, Saudi Arabia

**Keywords:** 3D printed resin, ZrO_2_ nanoparticles, denture base PMMA, mechanical testing, reinforcement

## Abstract

Due to the low mechanical performances of 3D-printed denture base resins, ZrO_2_ nanoparticles (ZrO_2_NPs) were incorporated into different 3D-printed resins and their effects on the flexure strength, elastic modulus, impact strength, hardness, and surface roughness were evaluated. A total of 286 specimens were fabricated in dimensions per respective test and divided according to materials into three groups: heat-polymerized as a control group and two 3D-printed resins (NextDent and ASIGA) which were modified with 0.5 wt.%, 1 wt.%, 3 wt.%, and 5 wt.% ZrO_2_NPs. The flexure strength and elastic modulus, impact strength, hardness, and surface roughness (µm) were measured using the three-point bending test, Charpy’s impact test, Vickers hardness test, and a profilometer, respectively. The data were analyzed by ANOVA and Tukey’s post hoc test (α = 0.05). The results showed that, in comparison to heat-polymerized resin, the unmodified 3D-printed resins showed a significant decrease in all tested properties (*p* < 0.001) except surface roughness (*p* = 0.11). In between 3D-printed resins, the addition of ZrO_2_NPs to 3D-printed resins showed a significant increase in flexure strength, impact strength, and hardness (*p* < 0.05) while showing no significant differences in surface roughness and elastic modulus (*p* > 0.05). Our study demonstrated that the unmodified 3D-printed resins showed inferior mechanical behavior when compared with heat-polymerized acrylic resin while the addition of ZrO_2_NPs improved the properties of 3D-printed resins. Therefore, the introduced 3D-printable nanocomposite denture-base resins are suitable for clinical use.

## 1. Introduction

The geriatric population has increased due to advancement in the health sector and expertise that have resulted in a rise in life expectancy [1]. Complete edentulism is considered one of the most common oral health problems affecting the geriatric population [2,3]. Although there are many methods of treatment, complete dentures are the preferred choice [4,5]. The materials used in the fabrication of complete removable dentures require certain characteristics to be accepted in the oral cavity, such as adequate strength within the minimum acceptable clinical ISO recommendation [6]. Polymethyl methacrylate (PMMA) is the most common denture-base material used in the manufacturing of complete dentures. The advantages that make PMMA the material of choice include its low cost, high aesthetics, good stability in the mouth, biocompatibility, and ease of fabrication and repair [7,8,9]. However, PMMA shows low strength, polymerization shrinkage, great susceptibility to microbial colonization, and low resistance to wear in an aqueous environment [10,11,12].

Attempts have been made to overcome these drawbacks, reinforcement being one of these [13,14,15,16]. The use of nanoparticles (NPs) was suggested to improve the properties of PMMA denture base [17]. The main motivation for NP implementation in nanomedicine was attributed to NPs’ synthesis process, which is sustainable, safe, and economical [18]. In previous studies, both zirconia (Zr) and zirconium dioxide (ZrO_2_) have been suggested, but ZrO_2_ has an advantage over Zr as it has a double bond with the acrylic after preforming through the silanization process [19]. However, Zr has a unique mechanical properties that can mimic the appearance of natural teeth and decrease peri-implant inflammatory reactions [20]. Among NPs, it was found that adding zirconium dioxide nanoparticles (ZrO_2_NPs) to the PMMA will enhance its characteristics [14]. ZrO_2_ is a biocompatible metal dioxide with high strength, fracture toughness, and surface hardness [21,22]. Moreover, ZrO_2_ has resistance to corrosion [23], thermal stability [24], and antibacterial and antifungal effects on *C. albicans* and *Aspergillus niger* [25]. Several studies found that incorporating specific concentrations of ZrO_2_NPs into PMMA increased the surface hardness, flexure strength, impact strength, and fracture toughness of the resin and enhanced it [15,16].

Digital manufacturing technologies have been largely used in dentistry in recent years. The fabrication of removable dentures can be performed at present using the technology of computer-aided design and computer-aided manufacture (CAD-CAM), which includes subtractive methods and additive methods, commonly known as 3D printing and rapid prototyping [26,27]. As a term, 3D printing is used to describe a manufacturing approach that builds objects one layer at a time [26,27]. Three-dimensional printing is a rapidly developing technology that has been tested by a lot of users. The reason for its rapid development is that it provides multiple advantages compared to conventional methods. It is also described as the key technology for the next industrial revolution [26,27].

Due to the revolution of 3D printing in dentistry, the fabrication of a complete denture base without the need of molds or cutting tools can be performed easily due to the ability of 3D-printing technologies in directly receiving CAD data and quickly creating a new digital model [28]. Another advantage of 3D printing is that the unnecessary materials are not consumed in the polymerization process because of the selective concentration of a laser beam on the selected part of the material [29]. In addition to the reduced time taken by procedures along with the lab work [30], it also shows advantages in terms of the improvement of tissue adaptation and the ease of duplicating existing dentures [31]. Even though PMMA is lower in cost than 3D-printed resins, both share some advantages, such as high aesthetics and biocompatibility with oral tissues. However, the 3D-printing method is more accurate compared to the conventional method, as it eliminates the errors made by lab technicians [7,8,9,31].

Even though the 3D-printing technology is the trend in fabricating complete removable denture bases at present, it still has some drawbacks related to its mechanical and physical properties [32,33,34,35]. Moreover, a study resulted in inferior double-bond conversion in 3D printing when compared to conventional acrylic resins [32]. Recent studies have shown that heat-polymerized resin displays superior elastic modulus, flexural strength, impact strength, and hardness values than 3D-printed resin, but showed inferior surface roughness [33,34]. Several studies showed the effect of additives on 3D-printed material [24,36,37,38]. A study that was conducted by Aati showed an improvement in provisional restorations modified with ZrO_2_ made by 3D-printed resin from a long-term perspective [24]. Mubarak et al. showed improvement in tensile strength, tensile modulus, and flexural strength after adding <1wt% of silver–titanium dioxide nanofiller to a 3D-printed material [36]. Chen et al. showed that a 3D-printed resin modified with cellulose nanocrystals and silver nanoparticles showed improvement in flexural strength and impact strength (0–0.1wt%) [37]. Another recent study was conducted to evaluate the surface and mechanical properties of 3D-printed resin with incorporated silicon dioxide nanoparticles, and the results showed general improvement in impact strength, flexural strength, and the hardness of the 3D-printed resin [38].

ZrO_2_NPs could be a suitable reinforcement method for 3D printing material [39]. To the best of the authors’ knowledge, no previous studies have investigated the effect of the addition of ZrO_2_NPs on the properties of 3D-printed resins for denture-base fabrication. Therefore, the aim of this study was to evaluate the effect of adding ZrO_2_NPs to 3D-printed denture-base resins on the flexure strength, elastic modulus, impact strength, hardness, and surface roughness in comparison to conventional heat-polymerized acrylic resin. The null hypothesis was that there was no effect on the flexure strength, impact strength, surface hardness, and surface roughness of 3D-printed material modified with ZrO_2_NPs.

## 2. Materials and Methods

The sample size of this in vitro study was counted using power analysis, using the World Health Organization formulae and determining a study power of 80%, a 5% level of significance and a 5% marginal error for the study.

### 2.1. Nanocomposites Mixture Preparation

One heat-polymerized PMMA and two 3D-printed resins Denture 3D+ (Denture 3D+, NextDent, Soesterberg, The Netherlands) and DentaBase (DentaBase; ASIGA, Sydney, Australia) were used in this study. ZrO_2_NPs (99.9% purity, Sigma-Aldrich, St. Louis, MO, USA) were added to 3D-printed resins in various concentrations (0.5wt%, 1wt%, 3wt%, 5wt%) while one group per resin remained without addition (unmodified group) [40]. Each group was printed at each percentage to have 286 specimens as a total number for the study, 110 for flexural strength (*n* = 10), 66 for impact strength (*n* = 6), and 110 for surface roughness and hardness (*n* = 10). The ZrO_2_NPs had an average granularity of 40 nm and a surface area of 9 m^2^/g, according to previous SEM and TEM analyses [41,42,43]. ZrO_2_NPs were treated with the silane-coupling agent TMSPM (Shanghai Richem International Co., Ltd. in Shanghai, China), as described in a previous study [44]. An electronic scale was used to weigh the silanized ZrO_2_NPs, which were then added in amounts of 0.5wt%, 1wt%, 3wt%, and 5wt% to both 3D-printing resins (“NextDent” and “ASIGA”). Fluid resins containing ZrO_2_NP were thoroughly mixed and stirred for 30 min as detailed in previous studies [13,45].

### 2.2. Specimens’ Preparations

According to the ISO 20795-1: 2013 standard [46], a rectangular bar with dimensions of (64 × 10 × 3.3 ± 0.2 mm) was designed to be used for a flexure-properties test, while other specimens were designed with dimensions of (50 × 6 × 4 mm) with a 1.2 mm edgewise V-notch, leaving a residual depth of 4.8 mm under the notch to measure the impact strength, whereas roughness and hardness test specimens were designed with dimensions of (15 × 2 mm).

Heat-polymerized PMMA specimens were fabricated from (Major.Base.20 MAJOR, Prodotti Dentari S.p.A. via Einaudi 23, 10,024 Moncalieri (TO)—ITALY) to be used as a control group. A metal mold was used to fabricate a wax pattern of the dimensions needed. The wax was invested in stone inside the flask to prepare it for de-waxing. A heat-polymerized acrylic resin was packed and then placed in a thermal polymerization unit (Wapo-Mat III, WASSERMANN Dental-Maschinen GmbH Rudorffweg 15–17 21,031 Hamburg, Germany) to be processed at 73 °C for 90 min then at 100 °C for an additional 30 min [47].

On the other hand, the 3D-printed specimens were designed using an open-source CAD system (123D design, Autodesk, version 2.2.14, California, USA), saved as an STL file, and printed by (NextDent 5100, NextDent, Soesterberg, The Netherlands) and (ASIGA MAXTM; ASIGA, Sydney, Australia), using 3D-printed machines, with the mentioned dimensions. Pure resin was placed on the machine (LC 3D Mixer, NextDent, Soesterberg, and The Netherlands) to be mixed before adding the nanoparticles for a period of 120 min; after adding the nanoparticles and distributing the resin into several bottles with different concentrations, each bottle was shaken for 30 min before proceeding to the printing process. Each layer was printed with a 50 µm layer thickness and 90 degree orientation [48], then exposed to a UV light with an intensity of 405 nm to be polymerized. After printing, all the specimens were cleaned using isopropyl alcohol (99.9%), then immersed in a bowl containing glycerol. According to the manufacturer’s guidance, the post-curing process had a duration of 20 min for the ASIGA specimens (ASIGA Flash, ASIGA, Sydney, Australia) and 10 min for the NextDent specimens (LC-3DPrint Box, NextDent, Soesterberg, The Netherlands) [49].

All specimens were trimmed of excess resin using low-speed rotary instruments. Finishing was undertaken sequentially using silicon carbide grinding paper (800, 1500, and 2000 grit). All specimens (heat-polymerized and 3D-printed) were polished conventionally using a 0.050-μm suspension (Master Prep polishing suspension; Buehler GmbH) combined with a polishing cloth (TexMet C10in, 42-3210; Buehler GmbH, Düsseldorf, Germany ) and polishing machine (Metaserv 250 grinder-polisher; Buehler GmbH, Lake Bluff, IL, USA) in wet conditions [50]. All specimens were polished by only one operator, in order to ensure comparable pressure of the polishing tools on the specimens. The specimens were rinsed with water followed by coarse and fine rubber tips before subjecting them to a thermo-cycling process. A total of 5000 cycles were performed in the thermocycling machine (Thermocycler THE-1100/THE-1200, SD MECHATRONIK GMBH Miesbacher Str. 34 · 83,620 Feldkirchen-Westerham Germany) at a temperature between 5–55 °C with 30 s of dwell time and 5 s for dripping, to simulate the temperature changes occurring intraorally over half a year [51,52].

### 2.3. Testing Procedures

Before testing, the specimens were immersed in a water bath for 50 ± 1 h at 37 °C. Immediately following this, the specimens were removed from the water and placed in containers immersed in room-temperature water [53].

#### 2.3.1. Flexural Strength and Elastic Modulus

A three-point test was used to evaluate the flexure strength and elastic modulus. The load was applied at the center of the specimen until fracture. The fracture load (N) was recorded to calculate the flexural strength (MPa) using the equation FS = 3Fl/2bh^2^, as well as elastic modulus using the equation EM = SI^3^/(4bh^3^d) (MPa).

#### 2.3.2. Surface Morphology and Chemical Bonding (SEM and FTIR)

The fractured surfaces of PMMA and both groups (ASIGA and NextDent) were analyzed using a scanning electron microscope (SEM, FEI, Inspect S50, Czech Republic at 20 kV), evaluating the evenness of the distribution of ZrO_2_NPs within the resin matrix. The bonding of the specimens (ASIGA and NextDent groups) prepared under various ZrO_2_NP concentrations (0wt, 0.5wt, 1wt, 3wt, and 5wt%) were explored using Fourier transform infrared spectroscopy (FTIR) (Nicolet 6700, FTIR spectrometer). The specimens were scanned between 4000 and 400 cm^−1^ to obtain the FTIR spectra.

#### 2.3.3. Impact Strength

Impact strength was measured by Charpy’s impact test (Digital Charpy Izod impact tester, XJU 5.5; Jinan Hensgrand Instrument Co., Ltd., Jinan, China) [54,55]. Each specimen was horizontally positioned with a certain distance between the two fixed supports. A 500 g weight was then dropped at the mid-span of the specimen on the opposite side to the notch, and the value of the impact strength (kJ/m^2^) was digitally recorded.

#### 2.3.4. Hardness

Hardness was measured using a Vickers tester (Wilson Hardness; ITW Test and Measurement GmbH, Shanghai, China) and each specimen was subjected to a 50 N of load for 10 s on three different sites. The final hardness (VHN) value of each specimen was arithmetically calculated by obtaining the average of the three readings.

#### 2.3.5. Surface Roughness (Ra, µm)

A non-contact profilometer (Contour Gt-K1 optical profiler; Bruker Nano, Inc., Tucson, AZ, USA) was used for the measurement of the specimens’ surface roughness. The specimens were radially scanned 3 times at different points with 0.01 mm resolution and the average surface roughness (µm) for each specimen was calculated. Finally, to ascertain pit features, a software package was used to analyze the obtained images (Vision64; Bruker Nano, Coventry, UK).

##### Statistical Analysis

To test the data normality, the Shapiro–Wilk test was used, and insignificant results revealed that the data were normally distributed, hence parametric tests were used for data analysis. Therefore, a one-way ANOVA was used to test any significance in the variation in tested properties of the materials due to changes in the concentration levels. Furthermore, Tukey’s post hoc test was used for the pairwise comparison of means. In addition, a two-way ANOVA was used to test the combined effect of concentration levels and materials on the tested properties. *p*-values less than 0.05 were considered statistically significant.

## 3. Results

### Ftir Results

**Figure 1:**
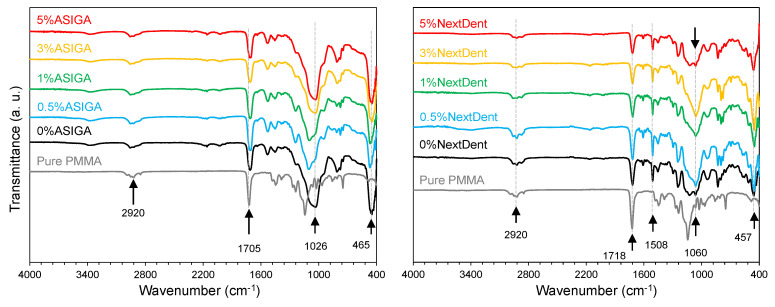
FTIR spectra of ASIGA and NextDent groups at different ZrO_2_ concentrations (0, 0.5, 1, 3, and 5 wt.%). The FTIR spectrum of pure PMMA specimen is also shown.

The FTIR results for ASIGA and NextDent at different ZrO_2_ concentrations (0 wt%, 0.5wt%, 1wt%, 3wt%, and 5wt%), along with pure PMMA, are displayed in Figure 1. By comparing the spectra, it is obvious that the bands of 3D-printed resins (ASIGA and NextDent) are different than those for pure PMMA, especially in the 1600 and 400 cm^−1^ range of the spectra, suggesting a different structure for the 3D-printed resins. For the ASIGA group, the strongest and widest bands appeared at ~1026 and ~465 cm^−1^ while ~1060 and ~457 cm^−1^ are the most intense bands for the NextDent resins. On the other hand, FTIR spectra showed some similar bands, highlighting the common characteristic features between 3D-printed resins (ASIGA and NextDent) and pure PMMA resin. The common bands of 3D-printed and pure PMMA matrices are attributed to CH_3_ and CH_2_ stretching vibrations (2920 cm^−1^) and carbonyl groups (1705 cm^−1^) in the PMMA chain [56,57]. The appearance of 1151 cm^−1^ bands confirmed C-O-C stretching vibrations of different modes. The FTIR spectra of pure PMMA and the two 3D-printed resins were found to be different from each other, suggesting that the chemical structure is different to some extent for ASIGA and NextDent groups compared to pure PMMA base denture. However, the comparison provides almost identical features and bands between the two groups (ASIG and NextDent), confirming the homogeneous dispersion of ZrO_2_ NPs into the 3D-printed resins.

**Figure 2:**
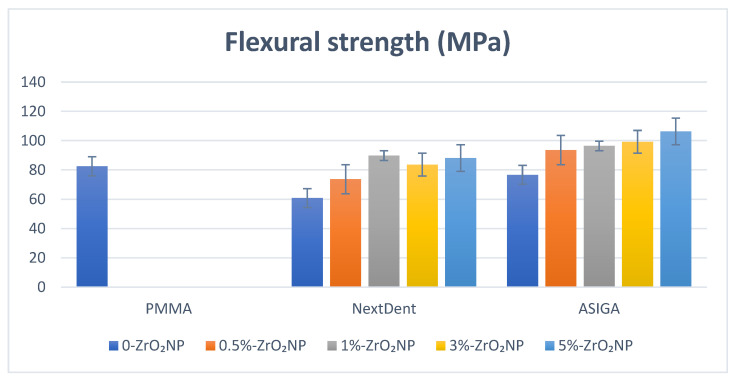
Flexural strength values and comparison between tested groups.

The mean and standard deviation and significance levels of all tested properties for NextDent and ASIGA are summarized in Table 1 and Table 2, respectively, along with Figure 2 and Figures 5–8. The highest mean of flexure strength was recorded for 1%-NextDent (89.8 ± 2.1 MPa) and the lowest was 0 wt.%-NextDent (60.8 ± 4.7 MPa). Tukey’s post hoc test showed a significant decrease in flexural strength of 0%-NextDent when compared with control (*p* < 0.05). With the addition of ZrO_2_NPs, the flexural strength was significantly increased compared to 0%-NextDent (*p* = 0.001). The highest flexural strength value was recorded with 5%-ASIGA (106.3 ± 16.9 MPa) and the lowest was 0%-ASIGA (76.7 ± 11.2 MPa). According to Tukey’s post hoc results, there were no significant differences between control and 0%-ASIGA (*p* = 0.09) while with the addition of ZrO_2_NPs, the flexural strength was significantly increased compared to 0%-ASIGA (*p* = 0.001) except for 0.5%-ASIGA, and this increase was concentration dependent.

**Figure 3:**
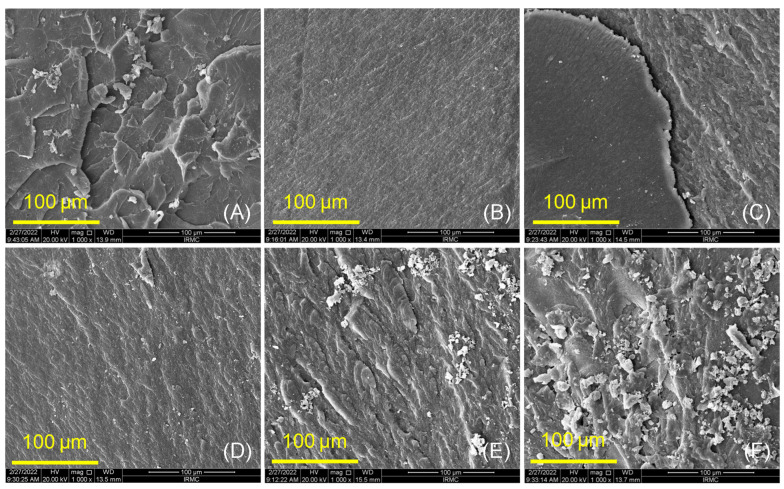
SEM images of ASIGA group at different concentrations of ZrO_2_NPs (0, 0.5, 1, 3, and 5 wt.%). (**A**) pure PMMA, (**B**) 0 wt.% ASIGA, (**C**) 0.5 wt.% ASIGA, (**D**) 1 wt.% ASIGA, (**E**) 3 wt.% ASIGA and (**F**) 5 wt.% ASIGA. The scale bars are 100 μm.

**Figure 4:**
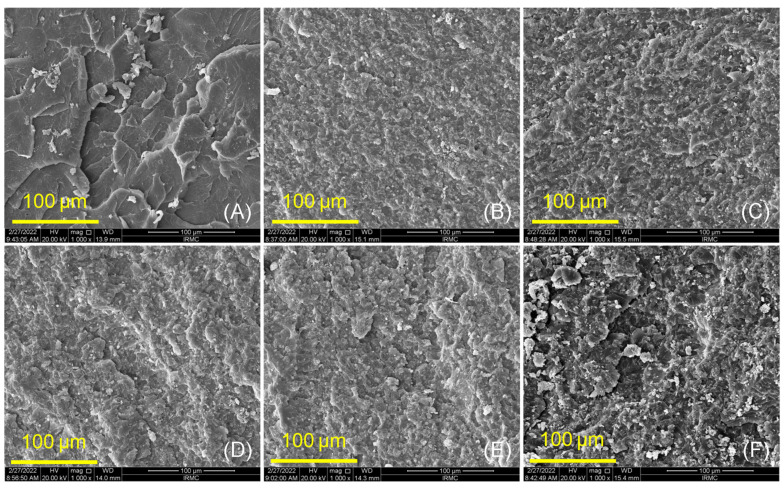
SEM images of NextDent group at different concentrations of ZrO_2_NPs (0, 0.5, 1, 3, and 5 wt.%). (**A**) pure PMMA, (**B**) 0 wt.% NextDent, (**C**) 0.5 wt.% NextDent, (**D**) 1 wt.% NextDent, (**E**) 3 wt.% NextDent and (**F**) 5 wt.% NextDent. The scale bars are 100 μm.

SEM analysis (Figure 3 and Figure 4) showed characteristic changes between control, unmodified, and ZrO_2_NP-modified 3D-printed denture-base resins (ASIGA and NextDent groups). At low concentrations, both materials showed a homogenous and good distribution of nanoparticles within the resin matrix (see Figure 3C,D and Figure 4C,D), while at high concentrations some clusters of the particles were displayed on the fractured surfaces (Figure 3F and Figure 4E,F). In the control specimens (Figure 3A and Figure 4A), random faint lamellae are observed, highlighting the brittle type of fracture. For unmodified NextDent and ASIGA specimens, the features of the resin displayed small lamellae with a smooth background (Figure 3B and Figure 4B), while these features were gradually changed from a smooth to a rough surface with the appearance of nanoparticles for the modified specimens, especially at higher concentration of ZrO_2_NPs (see Figure 3C–F and Figure 4C–F).

**Figure 5:**
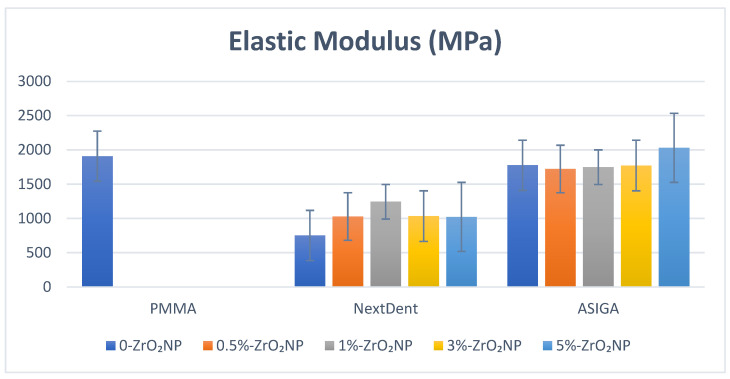
Elastic modulus values and comparison between tested groups.

One-way ANOVA analysis showed significant differences in elastic modulus for NextDent (*p* < 0.001) while showing no significant difference for ASIGA (*p* = 0.15). The highest mean of elastic modulus was recorded for NextDent (the control) (1909.4 ± 679.3 MPa) and the lowest was (0%) (751.8 ± 35.2 MPa). For NextDent, Tukey’s post hoc showed significant decreases in elastic modulus for 3D-printed groups compared with the control group (*p* < 0.001). The unmodified NextDent significantly showed the lowest elastic modulus compared with ZrO_2_NP-modified NextDent, while there was no significant difference between ZrO_2_NP-modified NextDent samples. The highest elastic modulus value was recorded with ASIGA (5%) (2031.2 ± 77.2 MPa) and the lowest was (0.5%) (1722.7 ± 122.1 MPa) (Figure 5).

**Figure 6:**
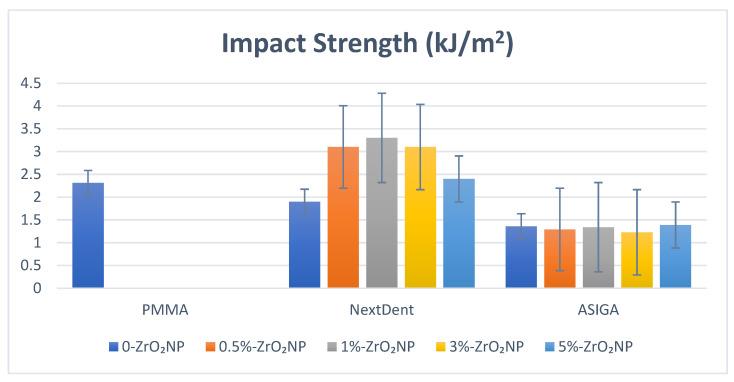
Impact strength values and comparison between tested groups.

For NextDent (Figure 6), the highest impact strength value was 1%-NextDent (3.3 ± 0.58 kJ/m^2^) and the lowest 0%-NextDent (1.9 ± 0.7 kJ/m^2^). For pairwise comparison, Tukey’s post hoc showed no significant difference between control and unmodified NextDent. With the addition of ZrO_2_NPs, the impact strength was increased significantly (*p* < 0.001) and the highest impact strength was recorded with 1%-NextDent (3.3 ± 0.58 kJ/m^2^). For ASIGA (Figure 6), the control significantly showed the highest impact strength value in comparison to all tested groups (*p* < 0.05) while no significant differences between unmodified and modified and ASIGA groups were observed.

**Figure 7:**
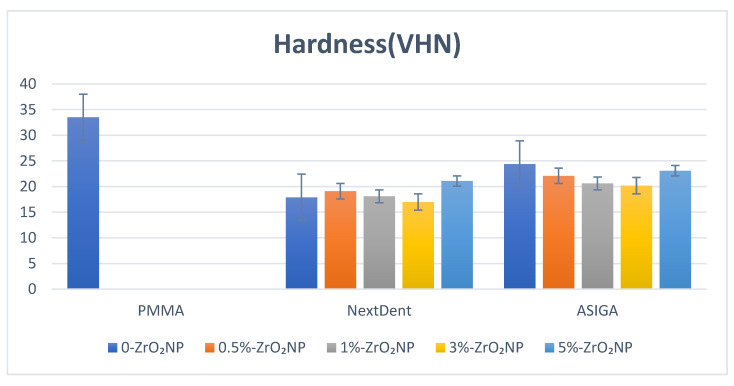
Hardness values and comparison between tested groups.

Heat-polymerized PMMA showed a significant increase in hardness compared with unmodified and ZrO_2_NP-modified NextDent and ASIGA (*p* < 0.001). Pairwise comparison of NextDent showed that 5%-NextDent significantly displayed the highest hardness value 21.1 ± 1.9 VHN). For ASIGA, no significant differences were found between unmodified and ZrO_2_NP-modified ASIGA. In between the modified groups, 5%-ASIGA showed the highest value (23.1 ± 2.2 VHN), while (3%) showed the lowest hardness value (20.2 ± 2.3 VHN) (Figure 7).

**Figure 8:**
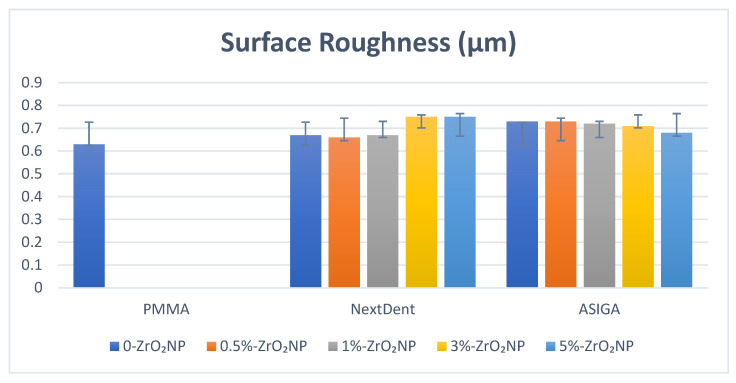
Surface roughness values and comparison between tested groups.

For NextDent, there are no significant differences in Ra between all test groups. The highest Ra value was recorded with 5%-NextDent (0.75 ± 0.12 µm) and the lowest was recorded with the control (0.63 ± 0.1 µm). For ASIGA, the control showed a significant decrease in Ra when compared with 0%-ASIGA and 0.5%-ASIGA which showed the highest Ra values (0.73 ± 0.03 µm and 0.73 ± 0.03 µm, respectively). In between the ZrO_2_NP-modified groups, as the concentration increased above 1%, the Ra decreased, while 5%-ASIGA showed the lowest Ra value (0.68 ± 0.13 µm) (Figure 8).

As we can see in Table 3, the 3D-printed specimens showed inferior results to PMMA. However, the addition of ZrO_2_NP enhanced the results in flexure strength by 47.6% and 38.5% when comparing the modified group with the unmodified for both NextDent and ASIGA, respectively. Furthermore, the results also showed that the modified specimens from both materials were better than PMMA by 8.8% and 28.8%. For the elastic modulus, the modified group showed superior results over both unmodified 3D-printed specimens by 65.5% and 14.3%. Nonetheless, only the modified ASIGA group showed better results than the PMMA. For the impact test, the modified NextDent group showed superior results when compared to the unmodified group and PMMA by 73.6% and 42.8%, respectively. However, the modified ASIGA group showed better results compared to the unmodified group by a percentage of 2.2% only. Although the modified group was smoother than the unmodified by 1.5% and 6.9%, PMMA was the most superior overall. Finally, for hardness, the modified NextDent group was 17.8% better when compared to the unmodified group.

## 4. Discussion

In this in vitro study, the effects of the addition of ZrO_2_NP on the properties of 3D-printed denture-base resins were tested. The null hypothesis was rejected due to the significant difference in values showed by 3D-printed resin compared to conventional heat-polymerized resin and ZrO_2_NP-modified 3D-printed resins. All the tested properties of NextDent had significant findings except for surface roughness. In contrast, ASIGA exhibited significant findings among all tested properties except for elastic modulus.

When the denture is in clinical use, it is subjected to an oral environment with thermal stresses, so specimens were subjected to thermal cycling for 5000 cycles, simulating the changes that happen in the oral cavity in half a year of clinical use [51]. The flexural strength of 3D-printed resin was tested because it is considered the primary mode of clinical failure [58]. Both groups of 3D-printed resin tested in the present study showed lower flexural strength values compared to heat-polymerized PMMA. The 3D-printed resin was printed layer by layer and polymerized via photo polymerization, which resulted in low flexural strength [59]. During specimen printing, the air entrapped within the resin fluid may have resulted in some void formation in and in between the printed layers. The presence of these voids affected the mechanical properties of the printed object [34]. Additionally, the polymerization techniques, in terms of the monomer conversion rate, could be another reason for the decreased flexural strength of 3D-printed resins [60].

Based on the findings of the present study, the flexural strength of 3D-printed resins was increased with the addition of ZrO_2_NPs, and this increase was concentration-dependent in the ASIGA resin. This is in agreement with previous studies that have investigated the effect of different nanoparticles on 3D-printed resins: denture-base resin modified with SiO_2_NPs [38] and provisional resins modified with nanodiamond [61] and ZrO_2_NP [24] showed an increase in the flexural strength for modified resins. This increase may be attributed to the homogenous distribution of ZrO_2_NPs within the resin matrix as confirmed by SEM finding that ZrO_2_NPs fill the interpolymeric spaces in addition to the transformation-toughening property of ZrO_2_NPs when load is applied, making the reinforced materials more resistant to crack propagation [38,61]. SEM displayed the ductile mode of fracture with ZrO_2_NPs, which required more energy for specimen fracture [61], confirming the effect of ZrO_2_NP on the strength of 3D-printed denture-base resins in addition to good bonding between ZrO_2_NPs and the resin matrix according to FTIR findings.

Many factors could lead to increased flexural strength, such as size, shape, concentration of filler, homogenous distribution within resin matrix, and the silanization process [17]. A previous study showed [39] ZrO_2_NPs added in different concentrations (3, 5, and 10 wt.%) and stated that as the concentrations increased the flexural strength increased up to 5% then an insignificant decrease occurred as the concentration increased up to 10%. The increase in flexural strength with ZrO_2_NP-modified ASIGA was concentration-dependent; as the concentrations increased, the flexural strength increased in ASIGA resin while in NextDent the highest was reported with 1%, without a significant difference in the 3 and 5% groups. This may be due to different compositions of 3D-printed resins. In the present study, the flexural strength of ZrO_2_NP-modified 3D-printed resins was higher than ISO recommendations (65MPa) in addition to some modified groups (1–5%) that exhibited higher values than the conventional PMMA resin we investigated in present study. That is considered a promising result, necessitating further investigations into long-term clinical effects.

Denture-base resins should have a high elastic modulus to prevent permanent deformation [62]. In this study, all 3D-printed resin showed lower elastic moduli compared to heat-polymerized PMMA. This can be explained by the combination of the reactivity of monomers of 3D-printed resin and the curing condition, which resulted in a lower degree of double-bond conversion when compared to conventional acrylic resins [53]. Another cause for the lower mechanical properties could be the weak interlayer bonding due to the degree of printing orientation [63,64]. According to this study, the addition of ZrO_2_NPs into the 3D-printed resin showed a significant increase in elastic modules for NextDent, which shows an agreement with Mangal’ study [61] who reported an increase in elastic modulus after adding nanodiamonds to a 3D-printed provisional resin. Similarly, Aati et al. [24] showed that the addition of ZrO_2_NPs increased elastic modulus significantly in provisional restorations. This increase can be related to the micron distance between the nanoparticles, which reduces the immobilization of the polymer chain. Moreover, the influence of ZrO_2_NPs on the strength of 3D-printed denture-base resins was proven by the modulus’s resilience, in which it absorbs the energy and stresses till deformity and fracture. [61]. Regarding the percentages of nanoparticles that were added, the ASIGA group showed no significance. However, in the NextDent group, all 3D-printed resins were less than the control group. Moreover, the unmodified group had the lowest value among all groups, although it started to increase as we increased the percentage of nanoparticles till it reached a peak value at 1%, where there was a significance difference between it and the 0%, following which the elastic modulus values started to drop again after more nanoparticles were added. In agreement with a previous study [24], adding different concentrations of ZrO_2_NP in (1, 2, 3, 4 and 5 wt.%) concentrations showed lower values than the control group, started to increase to a peak, and then started to drop gradually.

Accidentally dropping dentures is a common cause of denture fracture [65]. Therefore, the denture base should have materials incorporated into it that increase impact strength. The main cause of maxillary denture fracture is impact and fatigue forces while mandibular denture impact forces are responsible for 80% of fractures [66]. In the present study, heat-polymerized resin showed higher impact strength compared to unmodified 3D-printed resin. This decrease may be attributed the nature of the printing, where specimens were printed layer by layer, as discussed, for flexural strength. Hence, specimens were printed at 90 degrees; the load applied in parallel to the printing layer could be an explanation for the decreased impact strength [34].

In the current study, adding ZrO_2_NPs to 3D-printed resin resulted in a significant increase in impact strength. This finding was in line with Mangal [61] and Gad [38], who reported an improvement in impact strength when adding nanodiamond particles and SiO_2_NPs to 3D-printed resins, respectively. Concerning the concentration affect, as the concentration of ZrO_2_NPs increased, the impact strength increased. This improvement could be due to the fine particle size and appropriate ZrO_2_NP distribution, which could also lead to a prolonged crack length during the fracturing process. Energy absorption before fracture can be increased by increasing crack length before fracture [61]. In agreement with a previous study [38], adding ZrO_2_NPs in different concentrations (0.25 and 0.5 wt.%) increased the impact strength of 3D-printed material. The addition of ZrO_2_NPs to ASIGA did not result in an improvement in impact strength. Therefore, the results of impact strength must be interpreted with caution. These variations may be due to the difference in material composition, which needs further investigation to understand the behavior of ASIGA in terms of impact strength.

Hardness is a surface property that measures material resistance to localized plastic deformation induced by abrasion or mechanical indentation. A denture base that is fabricated with low-surface-hardness materials can be affected with mechanical brushing, resulting in color changes and plaque accumulation, which can reduce the denture’s integrity [53]. In this study, 3D-printed resins showed lower hardness values compared with heat-polymerized PMMA. The surface hardness could be adversely affected by the level of residual monomers, since their contents may have an impact on hardness [67]. In addition, water absorbed during thermal cycling acts as a plasticizer and decreases the mechanical performance of 3D-printed resins [34]. The addition of ZrO_2_NPs to the 3D-printed resins also resulted in an insignificant decrease in hardness values compared to unmodified resin. In contrast, Aati et al. [24] showed that the addition of ZrO_2_NPs increased the hardness significantly in provisional restorations. Similarly, Gad [38] reported that the incorporation of SiO_2_NPs into 3D-printed resin increased the hardness compared to unmodified 3D-printed resin. The material composition and type of nanoparticles and their concentration might be the cause of conflict. For each resin, the hardness values were insignificantly increased with the addition of 5% ZrO_2_NPs. This increase may be due to the saturation of printed specimens with high concentrations of ZrO_2_NPs, which resulted in the presence of NPs on the specimens’ surface in addition to the well-bonding silanated NPs and resin matrix [24].

Rough denture bases are easy to stain and provide a nest for microbial adherence leading to denture stomatitis [68]. Thus, the finishing and polishing of dental prostheses are required to reduce surface roughness [68]. In the present study, all 3D-printed resins showed higher surface roughness compared to heat-polymerized PMMA. The increased Ra may be attributed to the printing technology (layer by layer and printing orientation). In addition, the printing orientation (90°) resulted in more compact step-wise edges on the specimens’ surfaces, making the surface rougher between layers. On the other hand, ZrO_2_NP particle addition did not result in significant changes in Ra, and this accords with previous studies’ findings [24,38]. This explanation could be confirmed also with the addition of ZrO_2_NPs, which resulted in insignificant differences in Ra for the NextDent and ASIGA groups, with the exception of the unmodified and 0.5%-ASIGA groups. Hence, printing technology and printing parameters have a greater effect on Ra, and further investigations on different printing parameters’ effects on surface roughness are required.

According to the results of the present study, clinicians should consider the emergence of digitally produced dentures, although 3D-printed resin does have lower mechanical and surface properties compared to PMMA. Improving the 3D-printed resin by composition modification or strengthening is recommended to get the most out of the digital fabrication process. Furthermore, the addition of ZrO_2_NPs showed a promising result to overcome the drawbacks of 3D-printed resin properties. Moreover, the mechanical properties improved when using the introduced-nanocomposite 3D-printed resins, allowing for the clinical application of 3D-printed resin for denture-base fabrication. However, further investigations on the biocompatibility of nanocomposite 3D-printed resins are recommended.

The limitation of this study is using only one nanoparticle and one printing orientation. Furthermore, the specimen did not simulate the denture configuration, thermal cycling represented only a half year of intraoral use, and there was a lack of dynamic loading. Thus, this report suggests investigating different 3D-printed materials fabricated in a denture configuration and subjected to thermal and mechanical stresses similar to the intraoral environment, as well as using different nanoparticles, concentrations, and printing orientations.

## 5. Conclusions

Our 3D-printed resins showed inferior mechanical behavior and surface properties when compared with heat-polymerized acrylic resin. The addition of ZrO_2_NPs improved the properties of 3D-printed resins compared to heat-polymerized acrylic resins. Nanocomposite 3D-printed resins are suitable for clinical use and their limitations are decreasing. Flexural strength was concentration-dependent in the ASIGA group; the higher the concentration was, the higher the FS. On the other hand, NextDent did not show the same relationship. Unlike NextDent, the impact strength in the ASIGA group showed almost the same results for all concentrations added and all showed inferior impact strength compared to heat-polymerized acrylic resin. The addition of ZrO_2_NPs did not increase the elastic modulus or hardness in either group. On the other hand, both groups showed an increase in surface roughness.

## Figures and Tables

**Table 1:** Mean, SD, and significance of properties between groups concerning ZrO_2_NP concentration for NextDent.

ZrO_2_ Nanoparticles	Flexural Strength	Elastic Modulus	Impact Strength	Hardness	Surface Roughness
Control	82.5 (7.6) ^a^	1909.4 (679.3) ^a,b,c,d,e^	2.31 (0.2) ^a^	33.5 (3.8) ^a,b,c,d,e^	0.63 (0.1)
0%	60.8 (4.7) ^a,b,c,d,e^	751.8 (35.2) ^a,f^	1.9 (0.7) ^b,c,d^	17.9 (0.81) ^a^	0.67 (0.03)
0.5%	73.7 (5.6) ^b,f,g^	1029.1 (109.9) ^b^	3.1 (0.67) ^b^	19.1 (2.7) ^b^	0.66 (0.03)
1%	89.8 (2.1) ^c,f^	1244.3 (105.6) ^c,f^	3.3 (0.58) ^a,c^	18.1 (1.6) ^c^	0.67 (0.08)
3%	83.6 (5.9) ^d^	1034.2 (49.1) ^d^	3.1 (0.56) ^d^	17.0 (3.1) ^d,f^	0.75 (0.09)
5%	88.1 (15.6) ^e,g^	1022.9 (46.7) ^e^	2.4 (0.22)	21.1 (1.9) ^e,f^	0.75 (0.12)
*p*-value	0.000 *	0.000 *	0.000 *	0.000 *	0.11

* Statistically significant at 0.05 level of significance. Same small letters in each column show the significant difference between the pairs.

**Table 2:** Mean, SD, and significance of properties between groups concerning ZrO_2_NP concentration for the ASIGA.

ZrO_2_ Nanoparticles	Flexural Strength	Elastic Modulus	Impact Strength	Hardness	Surface Roughness
Control	82.5 (7.6) ^a,b^	1909.4 (679.3)	2.31 (0.2) ^a,b,c,d,e^	33.5 (3.8) ^a,b,c,d,e^	0.63 (0.1) ^a,b^
0%	76.7 (11.2) ^c,d,e,f^	1776.9 (99.6)	1.36 (0.17) ^a^	24.4 (4.5) ^a^	0.73 (0.03) ^a^
0.5%	93.6 (11.0) ^c^	1722.7 (122.1)	1.29 (0.12) ^b^	22.1 (1.9) ^b^	0.73 (0.03) ^b^
1%	96.4 (15.8) ^d^	1749.0 (66.3)	1.34 (0.1) ^c^	20.6 (1.9) ^c^	0.72 (0.08)
3%	99.2 (9.6) ^a,e^	1773.1 (59.8)	1.23 (0.27) ^d^	20.2 (2.3) ^d^	0.71 (0.09)
5%	106.3 (16.9) ^b,f^	2031.2 (77.2)	1.39 (0.3) ^e^	23.1 (2.2) ^e^	0.68 (0.13)
*p*-value	0.000 *	0.15	0.000 *	0.000 *	0.025 *

* Statistically significant at 0.05 level of significance. Same small letters in each column show the significant difference between the pairs.

**Table 3:** Summary of results based on ZrO_2_NP addition.

Materials	ZrO_2_NP	Tested Properties				
		FS	EM	SR	SH	IS
NextDent	Modified vs. PMMA	8.8%	−34.9%	−4.7%	−37.1%	42.8%
	Modified vs. Unmodified	47.6%	65.5%	1.5%	17.8%	73.6%
ASIGA	Modified vs. PMMA	28.8%	6.3%	−7.9%	−31.1%	−39.9%
	Modified vs. Unmodified	38.5%	14.3%	6.9%	−5.4%	2.2%

## Data Availability

Not applicable.

## References

[1] Slade G.D., Akinkugbe A.A., Sanders A.E. (2014). Projections of US edentulism prevalence following 5 decades of decline. J. Dent. Res..

[2] Cooper L.F. (2009). The current and future treatment of edentulism. J. Prosthodont..

[3] Al-Rafee M.A. (2020). The epidemiology of edentulism and the associated factors: A literature Review. J. Fam. Med. Prim. Care.

[4] Lee D.J., Saponaro P.C. (2019). Management of edentulous patients. Dent. Clin. N. Am..

[5] Anadioti E., Musharbash L., Blatz M.B., Papavasiliou G., Kamposiora P. (2020). 3D printed complete removable dental prostheses: A narrative review. BMC Oral Health.

[6] Diwan R., PatientsZarb G., Bolander C.L. (2004). Materials Prescribed in the Management of Edentulous Patients. Prosthodontic Treatment for Edentulous.

[7] Jaafar M. (2018). Review on poly-methyl methacrylate as denture base materials. Malays. J. Micros..

[8] Vojdani M., Bagheri R., Khaledi A.A. (2012). Effects of aluminum oxide addition on the flexural strength, surface hardness, and roughness of heat-polymerized acrylic resin. J. Dent. Sci..

[9] Meng T.R., Latta M.A. (2005). Physical properties of four acrylic denture base resins. J. Contemp Dent. Pract..

[10] Alla R., Raghavendra K.N., Vyas R., Konakanchi A. (2015). Conventional and contemporary polymers for the fabrication of denture prosthesis: Part I—Overview, composition and properties. Int. J. Appl. Dent. Sci..

[11] Gautam R., Singh R.D., Sharma V.P., Siddhartha R., Chand P., Kumar R. (2012). Biocompatibility of polymethylmethacrylate resins used in dentistry. J. Biomed. Mater. Res. B Appl. Biomater..

[12] Williams D.W., Chamary N., Lewis M.A., Milward P.J., McAndrew R. (2011). Microbial contamination of removable prosthodontic appliances from laboratories and impact of clinical storage. Br. Dent. J..

[13] Zhang X.-Y., Zhang X.-J., Huang Z.-L., Zhu B.-S., Chen R.-R. (2014). Hybrid effects of zirconia nanoparticles with aluminum borate whiskers on mechanical properties of denture base resin PMMA. Dent. Mater. J..

[14] Abushowmi T., AlZaher Z.A., Almaskin D.F., Qaw M.S., Abualsaud R., Akhtar S., Akhtar A.M., Al-Harbi F., Gad M.M., Baba N.Z. (2020). Comparative Effect of Glass Fiber and Nano-Filler Addition on Denture Repair Strength. J. Prosthodont..

[15] Gad M.M., Rahoma A., Al-Thobity A.M., ArRejaie A.S. (2016). Influence of incorporation of ZrO_2_ nanoparticles on the repair strength of polymethyl methacrylate denture bases. Int. J. Nanomed..

[16] Ahmed M.A., Ebrahim M.I. (2014). Effect of zirconium oxide nano-fillers addition on the flexural strength, fracture toughness, and hardness of heat-polymerized acrylic resin. World J. Nano Sci. Eng..

[17] Gad M.M., Fouda S.M., Al-Harbi F.A., Näpänkangas R., Raustia A. (2017). PMMA denture base material enhancement: A review of fiber, filler, and nanofiller addition. Int. J. Nanomed..

[18] Uzair B., Liaqat A., Iqbal H., Menaa B., Razzaq A., Thiripuranathar G., Rana N.F., Menaa F. (2020). Green and Cost-Effective Synthesis of Metallic Nanoparticles by Algae: Safe Methods for Translational Medicine. Bioengineering.

[19] Dallago M., Fontanari V., Torresani E., Leoni M., Pederzolli C., Potrich C., Benedetti M. (2018). Fatigue and biological properties of Ti-6Al-4V ELI cellular structures with variously arranged cubic cells made by selective laser melting. J. Mech. Behav. Biomed. Mater..

[20] Schünemann F.H., Galárraga-Vinueza M.E., Magini R., Fredel M., Silva F., Souza J.C., Zhang Y., Henriques B. (2019). Zirconia surface modifications for implant dentistry. Mater. Sci. Eng..

[21] Mallineni S.K., Nuvvula S., Matinlinna J.P., You C.K., King N.M. (2013). Bio compatibility of various dental materials in contemporary dentistry: A narrative insight. J. Investig. Clin. Dent..

[22] Kawai N., Lin J., Youmaru H., Shinya A., Shinya A. (2012). Effects of three luting agents and cyclic impact loading on shear bond strengths to zirconia with tribochemical treatment. J. Dent. Sci..

[23] El-Tamimi K.M., Bayoumi D.A., Ahmed M.M., Albaijan I., El-Sayed M.E. (2022). The Effect of Salinized Nano ZrO_2_ Particles on the Microstructure, Hardness, and Wear Behavior of Acrylic Denture Tooth Nanocomposite. Polymers.

[24] Aati S., Akram Z., Ngo H., Fawzy A.S. (2021). Development of 3D printed resin reinforced with modified ZrO_2_ nanoparticles for long-term provisional dental restorations. Dent. Mater..

[25] Gowri S., Gandhi R.R., Sundrarajan M. (2014). Structural, optical, antibacterial and antifungal properties of zirconia nanoparticles by biobased protocol. J. Mater. Sci. Technol..

[26] Dawood A., Marti B., Sauret-Jackson V., Darwood A. (2015). 3D printing in dentistry. Br. Dent. J..

[27] Kessler A., Hickel R., Reymus M. (2020). 3D Printing in Dentistry-State of the Art. Oper. Dent..

[28] Bassoli E., Gatto A., Iuliano L., Violante M.G. (2007). 3D printing technique applied to rapid casting. Rapid Prototyp. J..

[29] Bae E.J., Jeong I.D., Kim W.C., Kim J.H. (2017). A comparative study of additive and subtractive manufacturing for dental restorations. J. Prosthet. Dent..

[30] Ngo T.D., Kashani A., Imbalzano G., Nguyen K.T., Hui D. (2018). Additive manufacturing (3D printing): A review of materials, methods, applications and challenges. Compos. Part B Eng..

[31] Goodacre B.J., Goodacre C.J., Baba N.Z., Kattadiyil M.T. (2016). Comparison of denture base adaptation between CAD-CAM and conventional fabrication techniques. J. Prosthet. Dent..

[32] Alifui-Segbaya F., Bowman J., White A.R., George R., George R. (2019). Characterization of the double bond conversion of acrylic resins for 3D printing of dental prostheses. Compend. Contin. Educ. Dent..

[33] Perea-Lowery L., Gibreel M., Vallittu P.K., Lassila L.V. (2021). 3D-Printed vs. Heat-Polymerizing and Autopolymerizing Denture Base Acrylic Resins. Materials.

[34] Gad M.M., Fouda S.M., Abualsaud R., Alshahrani F.A., Al-Thobity A.M., Khan S.Q., Akhtar S., Ateeq I.S., Helal M.A., Al-Harbi F.A. (2021). Strength and Surface Properties of a 3D-Printed Denture Base Polymer. J. Prosthodont..

[35] Lee J., Belles D., Gonzalez M., Kiat-Amnuay S., Dugarte A., Ontiveros J. (2022). Impact strength of 3D printed and conventional heat-cured and cold-cured denture base acrylics. Int. J. Prosthodont..

[36] Mubarak S., Dhamodharan D., Kale M.B., Divakaran N., Senthil T., Wu L., Wang J. (2020). A Novel Approach to Enhance Mechanical and Thermal Properties of SLA 3D Printed Structure by Incorporation of Metal-Metal Oxide Nanoparticles. Nanomaterials.

[37] Chen S., Yang J., Jia Y.G., Lu B., Ren L. (2018). A study of 3D-printable reinforced composite resin: PMMA modified with silver nanoparticles loaded cellulose nanocrystal. Materials.

[38] Gad M.M., Al-Harbi F.A., Akhtar S., Fouda S.M. (2022). 3D-Printable Denture Base Resin Containing SiO_2_ Nanoparticles: An In Vitro Analysis of Mechanical and Surface Properties. J. Prosthodont..

[39] Hada T., Kanazawa M., Miyamoto N., Liu H., Iwaki M., Komagamine Y., Minakuchi S. (2022). Effect of Different Filler Contents and Printing Directions on the Mechanical Properties for Photopolymer Resins. Int. J. Mol. Sci..

[40] Zidan S., Silikas N., Alhotan A., Haider J., Yates J. (2019). Investigating the Mechanical Properties of ZrO_2_-Impregnated PMMA Nanocomposite for Denture-Based Applications. Materials.

[41] Gad M.M., Abualsaud R., Rahoma A., Al-Thobity A.M., Al-Abidi K.S., Akhtar S. (2018). Effect of zirconium oxide nanoparticles addition on the optical and tensile properties of polymethyl methacrylate denture base material. Int. J. Nanomed..

[42] Gad M.M., Abualsaud R., Rahoma A., Al-Thobity A.M., Akhtar S., Fouda S.M. (2022). Double-layered acrylic resin denture base with nanoparticle additions: An in vitro study. J. Prosthet. Dent..

[43] Alshahrani F.A., Gad M.M., Al-Thobity A.M., Akhtar S., Kashkari A., Alzoubi F., Yilmaz B. (2021). Effect of treated zirconium dioxide nanoparticles on the flexural properties of autopolymerized resin for interim fixed restorations: An in vitro study. J. Prosthet. Dent..

[44] Rahman H.A. (2015). The effect of addition nano particle ZrO_2_ on some properties of autoclave processed heat cure acrylic denture base material. J. Bagh. Coll. Dent..

[45] Ayad N.M., Badawi M.F., Fatah A.A. (2008). Effect of reinforcement of high-impact acrylic resin with zirconia on some physical and mechanical properties. Arch. Oral Res..

[46] International Organization for Standardization (ISO) (2019). Dentistry—Base Polymers—Part 1: Denture Base Polymers. ISO 20795-1:2013. https://www.iso.org/standard/62277.html/.

[47] Alzayyat S.T., Almutiri G.A., Aljandan J.K., Algarzai R.M., Khan S.Q., Akhtar S., Matin A., Gad M.M. (2021). Antifungal efficacy and physical properties of poly(methylmethacrylate) denture base material reinforced with SiO_2_ nanoparticles. J. Prosthodont..

[48] Unkovskiy A., Bui P.H., Schille C., Geis-Gerstorfer J., Huettig F., Spintzyk S. (2018). Objects build orientation, positioning, and curing influence dimensional accuracy and flexural properties of stereolithographically printed resin. Dent. Mater..

[49] Lin C.H., Lin Y.M., Lai Y.L., Lee S.Y. (2020). Mechanical properties, accuracy, and cytotoxicity of UV-polymerized 3D printing resins composed of Bis-EMA, UDMA, and TEGDMA. J. Prosthet. Dent..

[50] Kwon J.S., Kim J.Y., Mangal U., Seo J.Y., Lee M.J., Jin J., Yu J.H., Choi S.H. (2021). Durable Oral Biofilm Resistance of 3D-Printed Dental Base Polymers Containing Zwitterionic Materials. Int. J. Mol. Sci..

[51] Gale M.S., Darvell B.W. (1999). Thermal cycling procedures for laboratory testing of dental restorations. J. Dent..

[52] Silva C.D., Machado A.L., Chaves C.D., Pavarina A.C., Vergani C.E. (2013). Effect of thermal cycling on denture base and autopolymerizing reline resins. J. Appl. Oral Sci..

[53] Prpić V., Schauperl Z., Ćatić A., Dulčić N., Čimić S. (2020). Comparison of Mechanical Properties of 3D-Printed, CAD/CAM, and Conventional Denture Base Materials. J. Prosthodont..

[54] Dikbas I., Gurbuz O., Unalan F., Koksal T. (2013). Impact strength of denture polymethyl methacrylate reinforced with different forms of E-glass fibers. Acta Odontol. Scand..

[55] Mowade T.K., Dange S.P., Thakre M.B., Kamble V.D. (2012). Effect of fiber reinforcement on impact strength of heat polymerized polymethyl methacrylate denture base resin: In vitro study and SEM analysis. J. Adv. Prosthodont..

[56] Mujahid A., Choudhary T., Mehmood M., Irshad M., Hussain T., Bajwa S.Z., Ahmad M.N. (2019). Nickel sulfide nanoparticles incorporated poly (methyl methacrylate)-zirconia membranes for ultra deep desulfurization of dibenzothiophene. MRS Adv..

[57] Ajibade P.A., Mbese J.Z. (2014). Synthesis and characterization of metal sulfides nanoparticles/poly (methyl methacrylate) nanocomposites. Int. J. Polym. Sci..

[58] Chitchumnong P., Brooks S.C., Stafford G.D. (1989). Comparison of three- and four-point flexural strength testing of denture-base polymers. Dent. Mater..

[59] Reymus M., Lümkemann N. (2019). Stawarczyk: 3D-printed material for temporary restorations: Impact of print layer thickness and post-curing method on degree of conversion. Int. J. Comput. Dent..

[60] Zeidan A.A.E., Sherif A.F., Baraka Y., Abualsaud R., Abdelrahim R.A., Gad M.M., Helal M.A. (2022). Evaluation of the Effect of Different Construction Techniques of CAD-CAM Milled, 3D-Printed, and Polyamide Denture Base Resins on Flexural Strength: An In Vitro Comparative Study. J. Prosthodont..

[61] Mangal U., Seo J.Y., Yu J., Kwon J.S., Choi S.H. (2020). Incorporating Aminated Nanodiamonds to Improve the Mechanical Properties of 3D-Printed Resin-Based Biomedical Appliances. Nanomaterials.

[62] Gad M.M., Fouda S.M., ArRejaie A.S., Al-Thobity A.M. (2019). Comparative Effect of Different Polymerization Techniques on the Flexural and Surface Properties of Acrylic Denture Bases. J. Prosthodont..

[63] Shim J.S., Kim J.E., Jeong S.H., Choi Y.J., Ryu J.J. (2020). Printing accuracy, mechanical properties, surface characteristics, and microbial adhesion of 3D-printed resins with various printing orientations. J. Prosthet. Dent..

[64] KEßLER A., Hickel R., Ilie N. (2021). In vitro investigation of the influence of printing direction on the flexural strength, flexural modulus and fractographic analysis of 3D-printed temporary materials. Dent. Mater. J..

[65] Faot F., Costa M.A., Cury A.A., Garcia R.C.R. (2006). Impact strength and fracturemorphology of denture acrylic resins. J. Prosthet. Dent..

[66] Sasaki H., Hamanaka I., Takahashi Y., Kawaguchi T. (2016). Effect of long-term water immersion or thermal shock on mechanical properties of high-impact acrylic denture base resins. Dent. Mater. J..

[67] Al-Dwairi Z.N., Ebrahim A.A.A.H., Baba N.Z. (2022). A Comparison of the Surface and Mechanical Properties of 3D Printable Denture-Base Resin Material and Conventional Polymethylmethacrylate (PMMA). J. Prosthodont..

[68] Gad M.M., Fouda S.M. (2020). Current perspectives and the future of Candida albicans-associated denture stomatitis treatment. Dent. Med. Probl..

